# Prevalence and bother of lower urinary tract symptoms and overactive bladder in Poland, an Eastern European Study

**DOI:** 10.1038/s41598-020-76846-0

**Published:** 2020-11-13

**Authors:** Mikolaj Przydacz, Tomasz Golabek, Przemyslaw Dudek, Marek Lipinski, Piotr Chlosta

**Affiliations:** 1 Department of Urology, Jagiellonian University Medical College, Krakow, Poland; 2 Department of Urology, Medical University of Lodz, Lodz, Poland

**Keywords:** Urology, Epidemiology

## Abstract

The prevalence of lower urinary tract symptoms (LUTS) and overactive bladder (OAB) has been measured by population-based investigations in many parts of the world. However, data are lacking for Eastern Europe, and there has not been any large population-representative study in any country of this region. Therefore, the aim of this study was to evaluate the prevalence and associated bother of LUTS and OAB in a population-representative sample of persons aged ≥ 40 years in Poland. This investigation was conducted as a computer-assisted telephone interview. The survey sample was stratified by age, sex, and place of residence to reflect the entire Polish population. LUTS and OAB were assessed by a standardized protocol based on the International Continence Society definitions and validated questionnaires. Of 6005 participants, 57% were women, and the mean age (range) was 60.7 (40–93) years. The prevalence of LUTS was 69.8% (men 66.2%; women 72.6%). There was no difference in prevalence between urban and rural areas. LUTS were often bothersome among men and women, but women were more likely to be bothered compared with men. There were also statistically significant correlations between the frequency and the bother intensity of each of the LUTS. The prevalence of OAB was higher in women (39.5%) than in men (26.8%), and OAB increased with age. Lastly, LUTS had detrimental effects on the quality of life because one third of the participants had concerns about their urinary-specific quality of life. This investigation was the first nationwide, population-representative epidemiological study of LUTS and OAB in an Eastern-European country. LUTS were highly prevalent, often bothersome, and had negative effects on the quality of life of men and women aged ≥ 40 years. Our findings are comparable with other epidemiologic studies of LUTS and OAB conducted in different regions of the world.

**Trial registration**: NCT04121936.

## Introduction

Lower urinary tract symptoms (LUTS) include storage, voiding, and post-micturition symptoms, whereas overactive bladder (OAB) syndrome is a subgroup of storage symptoms that includes urinary urgency, urge urinary incontinence, frequency, and nocturia^1^. Several large epidemiological studies have evaluated the prevalence and bother of LUTS, including OAB, in population-based analyses^2–4^. These studies reported that LUTS were highly prevalent and bothersome, LUTS affected over 60% of men and women, with some variability depending on study population, age, survey methodology, data collection, definition of LUTS, and culture or ethnicity.

However, data for LUTS are lacking for Central and Eastern Europe. Even in large-scale European epidemiological studies conducted to ascertain the prevalence of LUTS, countries from Central and Eastern Europe have not been included^2,3^. To date, no large population-representative study in any country of this region has reliably evaluated the prevalence of all LUTS and OAB using the definitions approved by the International Continence Society (ICS)^1^. These data are necessary to promote health, increase awareness, and reduce the burden of disease. Population estimates attract interdisciplinary frameworks for national health improvement programs instituted with appropriate allocation of resources by governments and healthcare systems.

Poland is the largest country in Central Europe^5,6^. By land area, Poland is the third largest in Eastern Europe, after Russia and Ukraine, and the farthest east of the European Union countries^7^. No study has been conducted in Poland to investigate the prevalence of any of LUTS or OAB at the general population level, despite a considerable need to identify the extent of these conditions to guide Polish health care policy and clinical practice. In addition, Poland possesses a somewhat unique set of demographics (i.e., supra-ethnic uniformity, ≥ 99% of residents of Caucasian race and ≥ 95% of residents of Polish identity^8^ and healthcare system (i.e., public and private sectors). Thus, it is of some interest to establish the prevalence of LUTS and OAB in Poland compared to their occurrence in less uniform populations^9^. Also, as Slavic people, Poles are culturally different from other European people, particularly Germanic and Romance people^10^. Until now, no reliable study on LUTS or OAB prevalence has been conducted in any predominantly Slavic country. Because some cultural norms such as lifestyle factors (e.g., diet) may affect health, the quality of data on prevalence and associated bother of LUTS may vary between countries and regions^2^. Additionally, with a relatively high number of people living in Polish rural regions, available data on LUTS and OAB prevalence may not be fully transferable to Poland because no population-representative analyses of LUTS or OAB prevalence have reported and compared outcomes for urban and rural areas. Consequently, our understanding of the prevalence and true burden of LUTS in Poland is extremely limited. Fortunately, the importance of population-based urological studies has gained attention in Poland and Central-Eastern Europe. This new focus is related mainly to the high prevalence of diverse LUTS and OAB among adults of both sexes in western countries and to the increasing awareness of the detrimental impact of LUTS and OAB on health-related quality of life. Therefore, the aim of this study was to evaluate, for the first time, the prevalence and bother of LUTS, including OAB, in a population-representative sample of adults aged ≥ 40 years in all geographical regions of Poland. The study was based on standardized symptom definitions provided by the ICS and on validated survey instruments.

## Methods

This investigation was a population-based cross-sectional analysis conducted to provide representative prevalence estimates (by age, sex, and place of residence) of LUTS and OAB in Poland. The research ethics committee of Jagiellonian University Medical College, Krakow, Poland approved the study (1072.6120.160.2019); in addition, the study was registered with ClinicalTrials.gov (NCT04121936). After a brief introduction about the study, all participants provided verbal informed consent before beginning the telephone interview. Participants could opt-out from the study at any moment. Standardized guidelines and well-established recommendations for reporting observational studies were followed^11^.

### Study design

We conducted computer-assisted telephone interviews (CATI) between 1 September and 30 December 2019. After considering the general applicability of surveys on population-representative samples in Poland, we chose a CATI system instead of direct interviews (limitations in stratifications for place of residence) and Internet surveys (limitations in stratifications for age, i.e., limited computer access or lack of computer skills by older persons)^12,13^. The survey was administered by Ipsos Poland, which represented itself with relevant quality certificates (PKJPA, PKJBI, OFBOR, ESOMAR)^14^. All interviewers underwent standardized training and regular quality-control checks. Pilot telephone-surveys and cognitive debriefing interviews (n = 100) were conducted before data collection to assess cultural and linguistic integrity, the ease of the CATI-survey format, and overall content validity with the conceptual interpretation of the questions. This pilot phase ensured that lay persons would correctly understand the survey questions.

Study participants were selected randomly by modified random-digit dialing. The most recent population census was employed as the basis for creating a target sample to ensure that the collected data would be representative of the general population^15^. Sample matching was used to construct population-representative sample of respondents. Before completing the questionnaires, the survey sample was stratified by age, sex, and place of residence (both for geographical regions and type/size of places of living) to reflect the entire Polish population^15^. Telephone numbers of potential study participants were stratified by zip code to ensure equal representation of all 16 states (voivodships)^15^. Both urban and rural areas were appropriately covered. Post-stratification weights were calculated to correct the amount of imbalance based on differences in response rates. The weights were computed by ranking the completed interviews to the marginals for the matching variables (i.e., age, sex, and place of residence) before all statistical analyses.

Eventually, the study included a representative pool of men and women, aged ≥ 40 years, living in all geographical regions of Poland. We excluded participants who had urinary tract infections within the preceding month and women who were either pregnant at the time of the survey or who had given birth within the preceding six months.

### Measures

For each participant, general demographic data were collected, including sex, age, level of education, employment status, and marital status. LUTS were assessed using a standardized protocol based on ICS definitions and evaluated storage symptoms (frequency, urgency, nocturia, urinary incontinence), voiding symptoms (intermittency, slow stream, hesitancy, straining, splitting/spraying, terminal dribble), and post-micturition symptoms (incomplete emptying, post-micturition dribble)^1^. We also included the International Prostate Symptom Score (IPSS), a widely used instrument that evaluates the severity of LUTS^16^, and the Overactive Bladder-Validated 8-question Screener (OAB-V8), a screening awareness tool that identifies patients with bothersome OAB symptoms^17^. All terms and questionnaires were validated in Polish. Participants were asked how often they experienced individual LUTS during the preceding month. Because Likert scales are superior to dichotomous responses and allow participants the opportunity to provide a real-life experience in LUTS^2^, a Likert-like scale was used with the following options: none (score 0), < 1 in 5 times (score 1), < half the time (score 2), about half the time (score 3), > half the time (score 4), or almost always (score 5). For every LUTS frequency response of at least ‘ < 1 in 5 times’, participants were asked about the degree of associated bother due to the particular LUTS (i.e., bother levels associated with each symptom were analysed by each bother question). Bother ratings were also assessed on a Likert-like scale: not at all (score 0), a little bit (score 1), somewhat (score 2), quite a bit (score 3), a great deal (score 4), or a very great deal (score 5). This approach made our results reliably comparable to studies that investigated the prevalence and burden of LUTS in other countries^2,18^.

### Objectives

The primary study objective was to estimate the prevalence of LUTS in men and women aged ≥ 40 years in Poland. For LUTS presence, previous investigators used either of two definitions that differed by the interval for estimating prevalence. Definition I: at least one storage, voiding, or post-micturition symptom with a Likert score 2–5, i.e., symptoms occurring less than half the time or more. Definition II: at least one storage, voiding, or post-micturition symptom with a Likert score 3–5, i.e., symptoms occurring half the time or more. To enable comparison with previous studies, we evaluated (separately) the prevalence of LUTS based on both definitions.

Secondary study objectives included the prevalence of specific LUTS, the bother of specific LUTS (LUTS were considered bothersome if they were rated at least quite a bit, i.e., Likert score 3–5), the prevalence of OAB (score ≥ 8 points from the OAB-V8), and overall assessment of severity of LUTS with an effect on quality of life (according to the IPSS).

### Statistics

All analyses were conducted separately for men and women. Descriptive statistics were used for demographic variables and initial data analysis. Chi-squared tests were used to evaluate differences in LUTS prevalence between the sexes and between age groups. In addition, linear association between frequency and bother of symptoms was evaluated using Spearman's rank correlation coefficient. Statistical significance was considered at *p* < 0.05. SPSS Statistics software (IBM Corporation, Armonk, NY, USA, version 24.0) was used to conduct data analysis.

For sample size calculation, we followed the methodology that was used in other studies of LUTS prevalence^19^. Therefore, the sample size was calculated based on the population age distribution and expected LUTS prevalence^20^. Age standardization depended on the recent census^15^. Assuming a 95% confidence interval, we calculated that a sample size of 4500 interviews in Poland would exceed the required sample size for estimating LUTS prevalence. After consult with two independent teams of epidemiologists who had healthcare backgrounds, and after analysing the recent Polish census with general recommendations for future population-representative studies, we decided to exceed the sample size and include 6000 participants to provide smaller margins of error without negative effects on statistical analyses^15^. With a national sample of 6,000, there was a 95% certainty that the overall survey results were within ± 1% of what they would have been had we polled the entire adult Polish population^15^. Because the response rate based on total contacts is typically 25–40% for a survey such as the one described here, and to reliably calculate post-stratification weights, 24,900 contacts were made to obtain the 6000 respondents.

### Ethics approval

The study was performed in compliance with Good Clinical Practice and in accordance with the Declaration of Helsinki. The research ethics committee of Jagiellonian University Medical College, Krakow, Poland approved the study (1072.6120.160.2019); in addition, the study was registered with ClinicalTrials.gov (NCT04121936). Informed consent was provided by all participants.

### Consent to participate

All participants provided informed consent.

## Results

In all, 14,384 persons met the criteria to participate in the study. Twenty percent of respondents (2866) refused to participate and 18% (2658) did not complete the interview. After careful calculations of post-stratification weights to reliably represent the entire Polish population for age, sex, and place of residence, we analyzed 6005 respondents. There were more women than men (57% vs. 43%). The mean age was 59.7 ± 11.7 for men and 61.5 ± 11.3 for women. Given the large sample size, the respondents were categorized into five age groups, 40–49, 50–59, 60–69, 70–79, and ≥ 80 (20%, 25%, 31%, 18%, 6%, respectively; percent of all respondents). More respondents lived in urban areas than in rural regions (62% vs. 38%). Seventy-five percent of the participants had at least secondary education. Table 1 presents detailed demographic characteristics.

**Table 1:** Demographic characteristics of the study population (n = 6005).

	Men		Women		Total		*P* value
	n	%	n	%	n	%	
Study participants	2612	100	3393	100	6005	100	
**Age category**							0.01
40–49	632	24	584	17	1216	20	
50–59	628	24	841	25	1469	25	
60–69	787	30	1096	32	1883	31	
70–79	427	17	671	20	1098	18	
≥ 80	138	5	201	6	339	6	
**Place of residence**							< 0.001
City with more than 500,000 inhabitants	320	12	393	12	713	12	
City with 100,000–500,000 inhabitants	481	18	576	17	1057	17	
City with 20,000- 100,000 inhabitants	544	21	638	19	1182	20	
City with less than 20,000 inhabitants	339	13	455	13	794	13	
Rural	928	36	1331	39	2259	38	
**Education level**							< 0.001
Elementary	116	4	215	6	331	6	
Vocational	599	23	551	16	1150	19	
Secondary	955	37	1403	42	2358	39	
Higher	942	36	1224	36	2166	36	
**Employment status**							< 0.001
Employed ^a^	1221	47	1094	32	2315	38	
Unemployed	133	5	124	4	257	4	
Pensioner/Retired	1150	44	1948	57	3098	52	
Other ^b^	108	4	227	7	335	6	
**Marital status**							< 0.001
Single	279	11	222	6	501	8	
Married or living with a partner	1941	74	2208	65	4149	69	
Separated or divorced	188	7	264	8	452	8	
Widower	204	8	699	21	903	15	

^a^This includes individuals who were employed, self-employed, owners of own business/service/professional practice, or autonomous.

^b^This includes housewife/husband, stipendiary, and others (i.e., not in the above categories).

### Primary study objective

**Figure 1:**
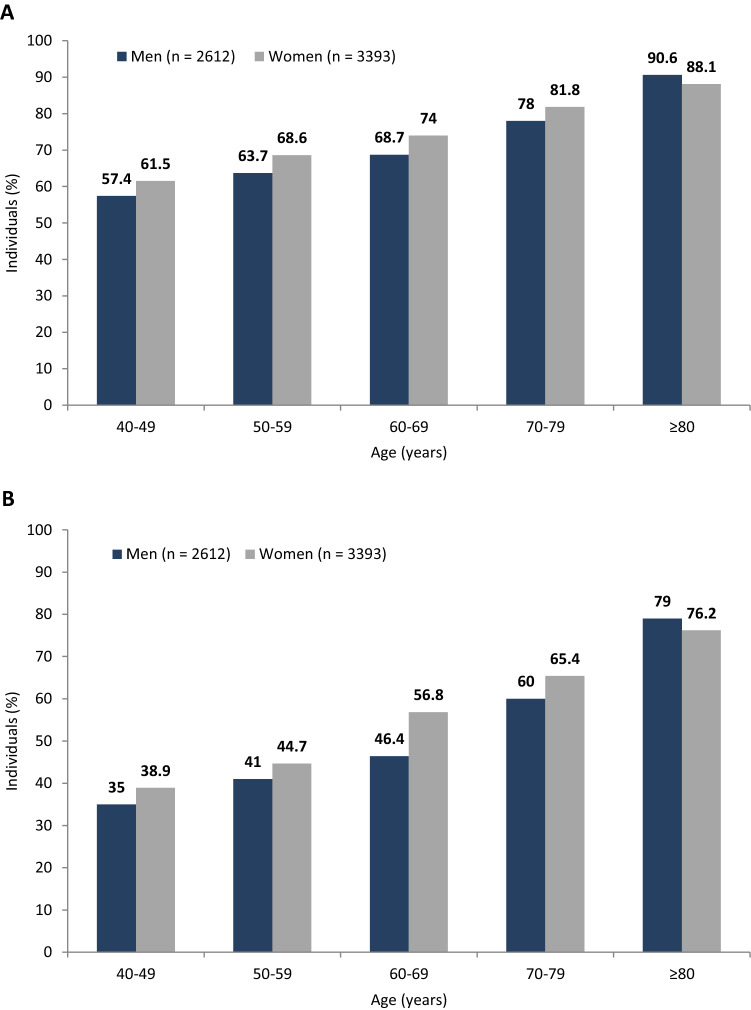
Prevalence of lower urinary tract symptoms based on the two study definitions: (**A**) definition I—symptoms occurring less than half the time or more; (**B**) definition II—symptoms occurring about half the time or more.

The primary study objective was to investigate the prevalence of LUTS. The prevalence of at least one LUTS at least ‘less than half the time’ (definition I) was 69.8% (men: 66.2%; women 72.6%; *p* < 0.001). The prevalence of at least one LUTS at least ‘half the time’ (definition II) was 50.4% (men: 46.2%; women: 53.5%; *p* < 0.001). For both men and women, the prevalence of LUTS increased with age Fig. 1, *p* < 0.001). In all age groups, except for ≥ 80, LUTS were more prevalent in women than in men. In the ≥ 80 age group, LUTS were more prevalent in men than in women (definition I: 90.6% vs. 88.1%; definition II: 79% vs. 76.2%). There was no difference in LUTS prevalence across geographical regions (voivodships) of Poland. There were also no significant associations between LUTS prevalence and urban/rural status.

### Secondary study objectives

#### Prevalence of specific LUTS

Nocturia, followed by frequency, both storage symptoms, were the most prevalent LUTS in general (Table 2). In a group of voiding symptoms, terminal dribble, followed by slow stream, were the most frequent. In a group of post-micturition symptoms, incomplete emptying was more common than post-micturition dribble.

**Table 2:** Prevalence of specific symptoms according to definition I (symptoms occurring less than half the time or more) and definition II (symptoms occurring about half the time or more) and associated bother in men and women.

	Men (n = 2612)						Women (n = 3393)					
	Symptom prevalence (definition I)		Symptom prevalence (definition II)		Prevalence of bother (at least quite a bit) ^a^		Symptom prevalence (definition I)		Symptom prevalence (definition II)		Prevalence of bother (at least quite a bit) ^a^	
	n	%	n	%	n	%	n	%	n	%	n	%
**Storage symptoms**												
Nocturia ^b^	805	30.8*	361	13.8	199	55.1	1213	35.8	554	16.3	341	61.6
Frequency	781	27.9*	480	18.4*	247	51.5	1116	32.9	720	21.1	421	58.5
Urgency	471	18*	259	9.9*	211	81.5	700	20.6	414	12.2	342	82.6
Urgency with fear of leaking	324	12.4***	194	7.4*	185	95.4*	643	18.9	391	11.5	309	79
Urge urinary incontinence	109	4.2***	61	2.3***	48	78.7	316	9.3	181	5.3	161	88.9
Stress urinary incontinence	53	2***	31	1.2***	26	83.9	415	12.2	248	7.3	231	93.1
Mixed urinary incontinence ^c^	55	2.1***	30	1.1***	26	78.8*	348	10.3	202	6	193	95.6
Leak for no reason	64	2.5**	31	1.2**	28	90.3	163	4.8	92	2.7	81	88
**Voiding symptoms**												
Intermittency	291	11.1	183	7	94	51.4	248	7.3	143	4.2	79	55.2
Slow stream	458	17.5*	271	10.4*	114	42*	321	9.5	163	4.8	85	52.1
Hesitancy	268	10.3*	133	5.1*	73	54.9*	173	5.1	80	2.4	34	42.5
Straining	179	6.9**	101	3.9*	64	63.4	89	2.6	39	1.1	24	61.5
Splitting/ spraying	258	9.9*	131	5	68	51.9	169	5	81	2.4	38	46.9
Terminal dribble	542	20.8**	348	13.3***	143	41.1	354	10.4	198	5.8	83	41.9
**Post-micturition symptoms**												
Incomplete emptying	315	12.1*	177	6.8	112	63.3	261	7.7	150	4.4	102	68
Post-micturition dribble	177	6.8*	87	3.3	56	64.4**	129	3.8	68	2	58	85.3

^a^Prevalence of bother was based on definition II.

^b^Nocturia was defined as two or more voids per night.

^c^Participants who reported both urge and stress urinary incontinence symptoms were classified as having mixed urinary incontinence.

*p ≤ 0.05, men versus women.

**p ≤ 0.01, men versus women.

***p ≤ 0.001, men versus women.

Considering ICS symptom groups overall (storage, voiding, post-micturition), we found that the storage symptom group was the most prevalent (definition I: 54.1% of men and 68.5% of women; definition II: 39.2% of men and 50.6% of women). Within ICS-specific symptom groups, storage symptoms were more prevalent in women than in men, whereas voiding symptoms and post-micturition symptoms were more prevalent in men than in women. Nocturia and frequency affected 35.8% and 32.9% of women, respectively, whereas 30.8% and 27.9% of men, respectively. Terminal dribble and incomplete emptying were present in 20.8% and 12.1% of men and in 10.4% and 4.4% of women, respectively.

**Figure 2:**
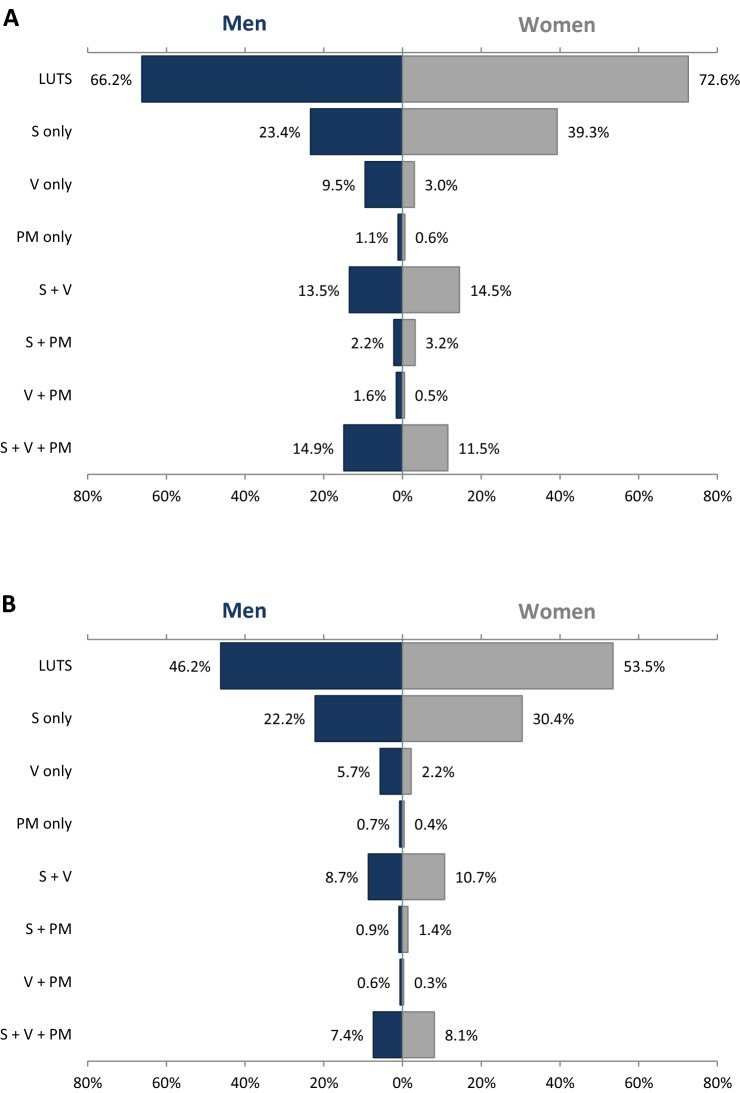
LUTS subtypes (storage, voiding, post-micturition, and combinations) in men and in women according to the two study definitions: (**A**) definition I (symptoms occurring less than half the time or more); (**B**) definition II (symptoms occurring about half the time or more). *LUTS* lower urinary tract symptoms; *PM* post-micturition symptoms; *S* storage symptoms; *V* voiding symptoms.

Further analysis of combinations of ICS symptom groups showed that a group of storage symptoms alone (i.e., participants who reported at least one storage symptom without any voiding or post-micturition symptoms) was the most common LUTS subtype in both men (23.4%) and women (39.3%) (Fig. 2). For men, the second most common group of ICS symptoms was the combination of storage, voiding, and post-micturition symptoms (14.9%), followed by a group of storage plus voiding symptoms (13.5%). For women, the second most common group was a group of storage plus voiding symptoms (14.5%), followed by the combination of all three groups (11.5%). More than 40% of all participants with LUTS had more than one symptom subtype, i.e., combination of at least two symptoms, each from a different ICS symptom group.

#### Bothersomeness of specific LUTS

In men, the most bothersome symptoms were urgency with fear of leaking, leak for no reason, and stress urinary incontinence (Table 2). The most bothersome symptoms among women were mixed urinary incontinence, stress urinary incontinence, and urge urinary incontinence. Overall, in both men and women, storage symptoms were more bothersome than voiding or post-micturition symptoms.

We found statistically significant correlations between the frequency of each of LUTS and intensification of their bother (i.e., the more frequent the occurrence of the symptoms, the more severe the bother, Table 3). The highest correlation coefficients were observed with storage symptoms (six of them met the correlation coefficient threshold of 0.8) compared with voiding symptoms (one of them met the correlation coefficient threshold of 0.8). The lowest correlation coefficient was observed for splitting/spraying (men: 0.619, women: 0.669) and the highest for leak for no reason (men: 0.838; women: 0.922).

**Table 3:** Correlations between the frequency of each LUTS and intensification of their bother.

	Sex		
	Men (n = 2612) r_S_*		Women (n = 3393) r_S_*
**Storage symptoms**			
Nocturia^a^	0.622	0.677	
Frequency	0.648	0.699	
Urgency	0.765	0.816	
Urgency with fear of leaking	0.783	0.832	
Urge urinary incontinence	0.841	0.898	
Stress urinary incontinence	0.835	0.882	
Mixed urinary incontinence^b^	0.833	0.878	
Leak for no reason	0.838	0.922	
**Voiding symptoms**			
Intermittency	0.655	0.699	
Slow stream	0.678	0.723	
Hesitancy	0.701	0.752	
Straining	0.771	0.842	
Splitting/spraying	0.619	0.669	
Terminal dribble	0.641	0.709	
**Post-micturition symptoms**			
Incomplete emptying	0.754	0.774	
Post-micturition dribble	0.837	0.889	

r_S_ Spearman's rank correlation coefficient (ranging from -1 indicating very strong negative association to + 1 indicating very strong positive association).

^a^Nocturia was defined as two or more voids per night.

^b^Participants who reported both urge and stress urinary incontinence symptoms were classified as having mixed urinary incontinence.

*p < 0.001 for all correlations.

### Prevalence of OAB

The prevalence of OAB as investigated with the OAB-V8 questionnaire (score ≥ 8 points) was 26.8% in men and 39.5% in women (Table 4). There was a statistically significant relationship between the OAB prevalence and sex (*p* < 0.01). Further, in both men and women, there was a significant correlation for increasing OAB prevalence with increasing age (*p* < 0.01, Table 4). OAB wet (i.e., defined as a score ≥ 8 points from the OAB-V8 and concomitant urge urinary incontinence occurring less than half the time or more) was more common in women (30.7% of all women with OAB) than in men (13.2% of all men with OAB).

**Table 4:** Data from the OABV8 (prevalence of OAB) and the IPSS (prevalence and severity of LUTS) questionnaires completed by men and women.

	Sex						
	Men		Women		Total		P value
	n	%	n	%	n	%	
**OAB-V8**							
OAB-V8 score ≥ 8 (all participants)	701	26.8	1340	39.5	2041	33.9	< 0.01
**Age category**							< 0.01
40–49	86	13.6	164	28.1	250	20.6	
50–59	141	22.5	312	37.1	453	30.8	
60–69	247	31.4	450	41.1	697	37	
70–79	160	37.5	308	45.9	468	42.6	
≥ 80	67	48.6	106	52.7	173	51	
**IPSS**							
Category (defined by the IPSS score)	2612	100	3393	100	6005	100	*p* = 0.28
None (score 0)	326	12.5	357	10.5	683	11.4	
Mild (score 1–7)	1619	62	2337	68.9	3956	65.9	
Moderate (score 8–19)	571	21.8	649	19.1	1220	20.3	
Severe (score 20–35)	96	3.7	50	1.5	146	2.4	

Prevalence according to definition I (symptoms occurring less than half the time or more).

### Overall assessment of LUTS severity with effects on quality of life

On the basis of the IPSS categories (none, mild, moderate, severe), we found that most of the participants had mild symptoms (62% of men and 68.9% of women; Table 4). In general, the prevalence and severity of symptoms investigated with IPSS were similar for men and women (*p* = non-significant).

Responses to IPSS question #8 (‘If you were to spend the rest of your life with your urinary condition just the way it is now, how would you feel about that?’) demonstrated that LUTS had negative effects on the quality of life. In a group of patients with LUTS at least ‘less than half the time’ (definition I), 27.3% of the participants (23.8% of men and 29.8% of women; *p* < 0.01) were ‘mixed’, ‘mostly dissatisfied’, ‘unhappy’, or ‘terrible’. In a group of respondents with LUTS at least ‘half the time or more’ (definition II), 41.7% of the participants (37.7% of men and 44.5% of women; *p* < 0.01) were ‘mixed’, ‘mostly dissatisfied’, ‘unhappy’, or ‘terrible’.

## Discussion

This cross-sectional analysis was the first population-representative epidemiological study of LUTS and OAB in an Eastern European country. Until now, a population-representative analysis of LUTS and OAB has not been conducted in any country of Central or Eastern Europe. To our knowledge, this study is also the first to evaluate the general population for the prevalence of any of LUTS in Poland. We performed the study across all geographical regions of the country in both urban and rural areas. The data provide reliable, valid, and consistent estimates of LUTS prevalence and symptom-specific bother. We showed that LUTS were highly prevalent and affected more women than men, with symptoms occurring ‘less than half the time or more’ in 66.2% of men and 72.6% of women and symptoms occurring ‘half the time or more’ in 46.2% of men and 53.5% of women. For both men and women, the prevalence of LUTS increased with advancing age.

The prevalence of LUTS has been estimated in several large population-based studies from various regions of the world. In Western Europe and North America, the Epidemiology Urinary Incontinence and Comorbidities (EPIC) study, a telephone survey in Canada, Germany, Italy, Sweden, and the UK (n = 19,165), reported the prevalence of LUTS of 62.5% in men and 66.6% in women^3^. Further epidemiological analysis in this area, the Epidemiology of Lower Urinary Tract Symptoms (EpiLUTS) study, an Internet-based population survey in Sweden, the USA, and the UK (n = 30,000), reported the prevalence of LUTS to be 72.3% for men and 76.3% for women^2^. In Asia, an Internet inquiry with participants from China, Taiwan, and South Korea (n = 8284) estimated the LUTS prevalence to be 62.8% for men and 59.6% for women^21^. In South America, the Brazil LUTS, a telephone interview conducted in five major cities of Brazil (n = 5184), showed the prevalence of LUTS of 69% in men and 82% in women^18^. Our observation (n = 6005) of LUTS in 69.8% of adults aged ≥ 40, affecting more women than men, appears broadly comparable with data from population-based studies performed in other countries and regions. Our data align with the results from the EPIC and EpiLUTS studies, i.e., symptoms were more prevalent in women than in men, and our data are contrary to Asian studies that documented greater LUTS prevalence in men than in women^21^. Further, an absolute difference between the sexes in our analysis (prevalence of LUTS of 72.6% in women and 66.2% in men) was also similar to the differences reported from western countries but, conversely, lower than the differences reported for South America (e.g., LUTS prevalence rates of 82% and 69% in women and men, respectively, in the Brazil LUTS study)^18^. Despite these slight differences, all the studies from various regions of the world have revealed a high overall prevalence of LUTS. Although environmental factors must be quite different, and peoples’ genetic backgrounds somewhat different, between the various places in which LUTS/OAB surveys have been conducted, we should admit that LUTS/OAB affect people worldwide. More importantly, it seems that LUTS/OAB occurrence may be largely independent of environmental or genetic influences. Our study conducted with a representative pool of a uniform population seems to further support this hypothesis.

Within the ICS symptom groups (storage, voiding, post-micturition), storage group was the most prevalent, and nocturia, followed by frequency, were the most prevalent symptoms, in both men and women. Nonetheless, co-existence of multiple symptoms was also common, and more than 40% of all the participants with LUTS in our study had more than one symptom subtype, i.e., combination of at least two symptoms, each from a different ICS symptom group. Similar patterns of ICS symptom group frequencies and symptom combinations were reported in other studies, which employed convergent methodology as the current analysis for estimating LUTS prevalence^3,18,22^. The overlap of symptoms has further implications, and it emphasizes that voiding symptoms are not the only LUTS in men and storage symptoms are not the only LUTS in women^23^. Therefore, our findings imply that LUTS can be approached broadly from the standpoint of symptoms without reference to disease or condition. LUTS are often related to bladder outlet obstruction. However, LUTS may also indicate other bladder and urinary tract irregularities, as well as non-urological aberrations^24^. Patients with symptoms from multiple categories force physicians to perform extensive and thorough diagnostic evaluation with a holistic approach for effective treatment because many, often overlapping, pathophysiological mechanisms are responsible for LUTS^23,25^.

Multiple studies have shown that LUTS may be highly bothersome. Some experts suggest that the aggravation caused by LUTS may be mostly related to patient perspective irrespective of how researchers define LUTS^2^. For both men and women, we found that storage symptoms were more bothersome than voiding or post-micturition symptoms. For men, the most bothersome LUTS were urgency with fear of leaking, leak for no reason, and stress urinary incontinence. For women, mixed urinary incontinence, stress urinary incontinence, and urge urinary incontinence were the most bothersome symptoms. These results were similar to findings from other studies. In the EpiLUTS, the Brazil LUTS and the study from Asia, storage symptoms were acknowledged as the most bothersome^2,18,21^. Urinary incontinence, leak for no reason, urgency with fear of leaking, urgency, and nocturia were generally pinpointed by participants as the most bothersome in those studies. Moreover, we uncovered strong connections between the frequency of each of LUTS and intensification of related bother. The severity of related bother was amplified as a function of the increased frequency of each LUTS. This observation of symptom frequency and related bother was also noted by other researchers in population-based analyses^2,3,18^. In our investigation, we observed the highest correlation coefficients with symptoms of urinary incontinence.

Because OAB is defined as a combination of symptoms that may represent OAB or coexistent conditions, the assessment of OAB prevalence may be highly ambiguous. Therefore, prevalences of OAB have been reported from as low as 2% to as high as 53%^26^. To make matters even more complex, expert panels suggest that only individuals who report bothersome OAB should be targeted for intervention^25^ because bother is related to treatment seeking^27^. As a result, different methods have been used to investigate OAB prevalence. In early studies, investigators estimated OAB prevalence from symptom combinations (e.g., urgency and urge urinary incontinence)^28,29^. Recent studies have been based on validated OAB screening instruments^18,21^. A patient’s notion of help seeking and perception of OAB treatment outcome are important factors for success, but these factors are subjective and highly individual^30^. Thus, validated instruments that measure relevant symptom burden may be optimal for population-based analyses. Estimates of OAB prevalence are more clinically accurate when investigators consider the degree of reported symptom bother in OAB^31^. Using the OAB-V8 questionnaire, we found an OAB prevalence of 26.8% in men and 39.5% in women. The OAB-V8 questionnaire has high sensitivity and specificity in recognizing OAB^20^, and it is accepted widely as a valid tool to estimate OAB prevalence in large population-based analyses^18,29,32^. Similar to our results obtained from the OAV-V8 questionnaire, we found women to have a greater frequency of urgency-related symptoms compared with men. The EpiLUTS study suggested that OAB was undertreated to comparable extents for men and women aged > 40 years in Sweden, the US, and the UK, with estimates ranging from 13 to 27% in men and 31% to 43% in women^22,33,34^. In contrast, the OAB prevalence reported from a Chinese cohort of adults ≥ 40 years old was 2.7% in men and 1.9% in women^35^, although a recent study from China, Taiwan, and Korea showed that OAB may affect as many as 21% of adults aged ≥ 40 years in this geographical area^36^. The wide discrepancy in OAB prevalence survey results has been well described and attributed to different variables^26^. When we consider differences in study populations and methods of measuring OAB, overall OAB prevalence in the current study appears comparable with international studies. Moreover, in our analysis, there was a significant association between OAB prevalence and age in both men and women, similar to international studies^37^. Because the prevalence of OAB increases with age, the number of people affected by OAB may further increase with population aging in the future^31,38^.

The IPSS is used globally, and it served as a benchmark in epidemiological studies of LUTS in the past^39–42^. The IPSS is used widely for both men and women because of its simplicity and reliability in assessing the severity of LUTS. Therefore, the IPSS can still be employed to compare LUTS prevalence across different analyses. In the EpiLUTS study, 26.3% of men and 34.5% of women aged ≥ 40 years had an IPSS score ≥ 8^2^. From the IPSS, the prevalence in Brazilian men ≥ 40 years was 21%, whereas the prevalence among women was estimated to be 24%^18^. Boyle et al. surveyed 4979 men and 3790 women in four cities in the UK, France, the Netherlands, and Korea. Moderate to severe symptoms (35 ≥ IPSS score ≥ 8) increased from age 40–79 years in both sexes and were 10.6–40.4% in men and 15.5–28.7% in women^43^. Despite the variability in these data, the prevalence of IPSS-defined LUTS we observed in adults aged ≥ 40 years (men, 25.5%,women, 20.6%) suggested broadly consistent prevalence of IPSS-defined LUTS in Poland as in other countries. Nevertheless, there is still a variability in IPSS-defined and ICS-defined LUTS prevalence. Different questionnaires (e.g., IPSS, ICS definitions) ask about LUTS differently and provide different response option formats. Respondent interpretations of the questions and response options might cause differences between studies, and translations into different languages can increase variability. The IPSS is the most used questionnaire for LUTS assessment, but it is limited by the inclusion of only seven questions, and the assessment of storage LUTS is particularly restricted. Further, IPSS does not provide any feedback for urinary incontinence, and the IPSS was developed prior to the emergence of the OAB concept. Because the IPSS determines the severity of symptoms, only respondents with more than a mild symptom are scored, instead of counting all respondents with any symptoms^28^. Therefore, IPSS use in population-based studies limits, and even underestimates, the prevalence and impact of individual LUTS^2,28^.

A strong point of our study was a large sample size, with well-balanced demographic characteristics. The variables were stratified by the recent census to ensure adequate representation of the entire population. The study covered all geographical regions of Poland, with proper proportions of urban and rural areas. Because our sample size exceeded 6,000 participants, the results were within ± 1% of statistical error for the national population. This exceedingly low margin of error makes our study one of the most accurate analyses of LUTS prevalence for a single country in the current literature. The study followed ICS terminology. Due to large sample size, our study also included, as a separate group, participants aged 80 years or more; these individuals have often been abandoned or underestimated in other population-based studies that considered participants aged 70 years or more as the top-most classification^3,18,26,28^. Authors of those studies often simply extrapolated prevalence for people aged 80 years or more^26^. We found that in men aged 80 years or more LUTS were more prevalent than in women, although in all other age groups LUTS were more prevalent in women than men. In other large-scale studies with the last age group of 70 years or more, the LUTS were more prevalent in women than in men regardless of age^3,18,28^. We included a wide variety of questions in the survey, and we employed well-established, validated diagnostic tools. Therefore, our study results, derived from current recommendations, provide a clear view of the prevalence and related bother of LUTS and OAB in Poland.

As with all studies that investigate a population, limitations included the use of self-reports without medical evaluation to measure LUTS. In addition, we relied on telephone interviews during which some individuals may not have provided accurate answers (especially with intimate information such as urinary incontinence). With cold-callings, respondents may not be fully open or honest. With surveys such as this, there is also a considerable risk of response bias related to participant attitude. Whereas some respondents with LUTS may have been likely to respond to a survey, those without LUTS may have been more likely to hang up or show less interest. Because this study was conducted in Poland, results may not be universally generalizable, e.g., for LUTS prevalence in urban and rural areas. However, similar LUTS prevalence has been reported in urban and rural populations in other countries^44^.

## Conclusions

This investigation was the first nationwide, population-representative epidemiological study of LUTS and OAB to be performed in Poland. LUTS were highly prevalent and often bothersome among men and women aged ≥ 40 years. Women were more likely to be affected than men. Although storage symptoms were more prevalent in women than in men and voiding or post-micturition symptoms were more prevalent in men than in women, specific symptoms and symptom groups were not attributed to only men or to only women. Coexistence of different symptoms was often observed. Our findings are consistent with other epidemiologic studies of LUTS conducted in different regions of the world.

## Data Availability

All data generated or analysed during this study are included in this published article.

## References

[1] Abrams P (2002). The standardisation of terminology of lower urinary tract function: report from the Standardisation Sub-committee of the International Continence Society. Neurourol. Urodyn..

[2] Coyne KS (2009). The prevalence of lower urinary tract symptoms (LUTS) in the USA, the UK and Sweden: results from the Epidemiology of LUTS (EpiLUTS) study. BJU Int..

[3] Irwin DE (2006). Population-based survey of urinary incontinence, overactive bladder, and other lower urinary tract symptoms in five countries: results of the EPIC study. Eur. Urol..

[4] Irwin DE, Kopp ZS, Agatep B, Milsom I, Abrams P (2011). Worldwide prevalence estimates of lower urinary tract symptoms, overactive bladder, urinary incontinence and bladder outlet obstruction. BJU Int..

[5] Organisation for Economic Co-operation and Development (OECD), Glossary of Statistical Terms [Internet]; Central and Eastern European Countries (CEECS), Publicated: 2001. https://stats.oecd.org/glossary/detail.asp?ID=303. Accessed 1 April 2020.

[6] World Bank Group, World Bank [Internet]; Poland At-A-Glance, Publicated: 2019. https://www.worldbank.org/en/country/poland. Accessed 1 April 2020.

[7] Publications Office of the European Union, EU Vocabularies [Internet]; Central and Eastern Europe, Publicated: 2019. https://op.europa.eu/en/web/eu-vocabularies/th-concept/-/resource/eurovoc/914. Accessed 1 April 2020.

[8] Glowny Urzad Statystyczny (GUS), Narodowy Spis Powszechny, Struktura narodowo-etniczna, językowa i wyznaniowa ludności Polski [National-ethnic, linguistic and religious structure of Poland. National Census of Population and Housing 2011] (in Polish); Publicated: 2015 [Cited: 2020 May] ISBN 978-83-7027-597-6.

[9] Jarczak J (2019). Mitochondrial DNA variability of the Polish population. Eur. J. Hum. Genet..

[10] Polish Scientific Publishers, Panstwowe Wydawnictwo Naukowe, PWN [Internet]; Slowianie, Publicated: 2019. https://encyklopedia.pwn.pl/haslo/Slowianie;3976552.html. Accessed 1 April 2020.

[11] von Elm E (2007). The strengthening the reporting of observational studies in epidemiology (STROBE) statement: guidelines for reporting observational studies. Lancet.

[12] Polska Agencja Rozwoju Przedsiebiorczosci (PARP), Raport metodologiczny z badan BKL [Internet]; Publicated: 2011 [Updated: 2020, Cited: 2020 May]. https://www.parp.gov.pl/component/publications.

[13] Na Strazy Sondazy, Uniwersytet Warszawski [Internet]; Publicated: 2013 [Cited: 2020 May]. https://nastrazysondazy.uw.edu.pl/metodologia-badan.

[14] Program Kontroli Jakosci Pracy Ankieterow (PKJPA), Organizacja Firm Badania Opinii i Rynku (OBFOR) [Internet]; Publicated: 2000 [Updated: 2019; Cited: 2020 May]. https://www.pkjpa.pl.

[15] Glowny Urzad Statystyczny (GUS), Narodowe Spisy Powszechne [Internet]; Publicated: 2012 [Cited: 2020 May]. https://stat.gov.pl/spisy-powszechne/.

[16] Barry MJ (1992). The American Urological Association symptom index for benign prostatic hyperplasia. The Measurement Committee of the American Urological Association. J. Urol..

[17] Coyne KS, Zyczynski T, Margolis MK, Elinoff V, Roberts RG (2005). Validation of an overactive bladder awareness tool for use in primary care settings. Adv. Ther..

[18] Soler R, Gomes CM, Averbeck MA, Koyama M (2018). The prevalence of lower urinary tract symptoms (LUTS) in Brazil: results from the epidemiology of LUTS (Brazil LUTS) study. Neurourol. Urodyn..

[19] Coyne KS (2009). Rationale for the study methods and design of the epidemiology of lower urinary tract symptoms (EpiLUTS) study. BJU Int..

[20] Llorente C (2010). New concepts in epidemiology of lower urinary tract symptoms in men. Eur. Urol. Suppl..

[21] Chapple C (2017). Prevalence of lower urinary tract symptoms in China, Taiwan, and South Korea: results from a cross-sectional, population-based study. Adv. Ther..

[22] Coyne KS (2011). The impact of overactive bladder on mental health, work productivity and health-related quality of life in the UK and Sweden: results from EpiLUTS. BJU Int..

[23] Sexton CC (2009). The overlap of storage, voiding and postmicturition symptoms and implications for treatment seeking in the USA, UK and Sweden: EpiLUTS. BJU Int..

[24] European Association of Urology (EAU), Non-Oncology Guidelines [Internet]; Management of Non-neurogenic Male LUTS, Publicated: 2020. https://uroweb.org/guideline/treatment-of-non-neurogenic-male-luts/. Accessed 1 April 2020.

[25] Corcos J., et al. CUA guideline on adult overactive bladder. *Canadian Urological Association journal = Journal de l'Association des urologues du Canada*. **11**, E142–E173 (2017). 10.5489/cuaj.4586 PMC5426936 28503229

[26] Tikkinen KA (2007). Is the prevalence of overactive bladder overestimated? A population-based study in Finland. PLoS ONE.

[27] Harpe SE, Szeinbach SL, Caswell RJ, Corey R, McAuley JW (2007). The relative importance of health related quality of life and prescription insurance coverage in the decision to pharmacologically manage symptoms of overactive bladder. J. Urol..

[28] Herschorn S, Gajewski J, Schulz J, Corcos J (2008). A population-based study of urinary symptoms and incontinence: the Canadian Urinary Bladder Survey. BJU Int..

[29] Irwin DE (2009). Prevalence, severity, and symptom bother of lower urinary tract symptoms among men in the EPIC study: impact of overactive bladder. Eur. Urol..

[30] Przydacz M, Golabek T, Chlosta P (2019). How to assess and predict success or failure of intra-detrusor injections with onabotulinumtoxinA. Adv. Clin. Exp. Med..

[31] Vaughan CP (2011). The prevalence of clinically meaningful overactive bladder: bother and quality of life results from the population-based FINNO study. Eur. Urol..

[32] Moreira ED, Jr, (2013). A population-based survey of lower urinary tract symptoms (LUTS) and symptom-specific bother: results from the Brazilian LUTS epidemiology study (BLUES). World J. Urol..

[33] Coyne KS (2011). National community prevalence of overactive bladder in the United States stratified by sex and age. Urology.

[34] Coyne KS, Sexton CC, Weinstein D (2009). The prevalence of OAB in the US, UK and Sweden: results from EpiLUTS. J. Urol..

[35] Wen JG (2014). The prevalence and risk factors of OAB in middle-aged and old people in China. Neurourol. Urodyn..

[36] Chuang YC (2019). Prevalence of overactive bladder in China, Taiwan and South Korea: results from a cross-sectional, population-based study. Low Urin. Tract Symptoms.

[37] Eapen RS, Radomski SB (2016). Review of the epidemiology of overactive bladder. Res. Rep. Urol..

[38] Irwin DE, Milsom I, Chancellor MB, Kopp Z, Guan Z (2010). Dynamic progression of overactive bladder and urinary incontinence symptoms: a systematic review. Eur. Urol..

[39] Hunter DJ, Berra-Unamuno A, Martin-Gordo A (1996). Prevalence of urinary symptoms and other urological conditions in Spanish men 50 years old or older. J. Urol..

[40] Pinnock C, Marshall VR (1997). Troublesome lower urinary tract symptoms in the community: a prevalence study. Med. J. Aust..

[41] Tuncay AF, Aygun C, Bilir N, Erkan I, Ozen H (2003). Prevalence of lower urinary tract symptoms in a community-based survey of men in Turkey. Int. J. Urol..

[42] Andersson SO, Rashidkhani B, Karlberg L, Wolk A, Johansson JE (2004). Prevalence of lower urinary tract symptoms in men aged 45–79 years: a population-based study of 40 000 Swedish men. BJU Int..

[43] Boyle P (2003). The prevalence of lower urinary tract symptoms in men and women in four centres. The UrEpik study. BJU Int..

[44] Egan KB (2015). Rural vs. urban disparities in association with lower urinary tract symptoms and benign prostatic hyperplasia in ageing men, NHANES 2001–2008. Int. J. Clin. Pract..

